# Multiparametric Coronary CT Angiography-Derived Imaging Biomarkers for Risk Stratification in Nonobstructive Coronary Artery Disease: Incremental Prognostic Value in Patients with Diabetes

**DOI:** 10.3390/tomography12070094

**Published:** 2026-06-25

**Authors:** Lei Chen, Hong Huang, Hao Tian, Wen-Yue Chen, Yong Wu, Hong-Yan Qiao, Jun Liu

**Affiliations:** 1 Department of Medical Imaging, Affiliated Hospital of Jiangnan University, No. 1000 Hefeng Road, Binhu District, Wuxi 214122, China; 2 Department of Emergency, Affiliated Hospital of Jiangnan University, Wuxi 214122, China; 9862022031@jiangnan.edu.cn

**Keywords:** diabetes mellitus, nonobstructive coronary artery disease, coronary computed tomography angiography, prognosis

## Abstract

**Background:** Patients with diabetes mellitus and nonobstructive coronary artery disease (NOCAD) may remain at increased cardiovascular risk despite the absence of flow-limiting stenosis. Quantitative coronary CT angiography (CCTA) enables comprehensive assessment of anatomical, functional, and inflammatory imaging biomarkers beyond luminal stenosis. This study aimed to evaluate the prognostic value of an automated multiparametric CCTA-derived imaging framework for risk stratification in patients with NOCAD, with exploratory assessment in those with diabetes mellitus. **Methods:** This retrospective single-center study included 485 patients with NOCAD who underwent CCTA between January 2020 and December 2021. Automated CCTA analysis was performed to quantify plaque burden, high-risk plaque features, CT-derived fractional flow reserve (CT-FFR), and perivascular fat attenuation index. The primary endpoint was major adverse cardiovascular events (MACE) during follow-up. Prognostic associations were assessed using Kaplan–Meier analysis, Cox regression, and hierarchical models. **Results:** During a median follow-up of approximately three years, MACE occurred in 56 patients. Patients with diabetes had a higher event rate than those without diabetes. Increased plaque burden, high-risk plaque features, elevated perivascular fat attenuation index, and reduced CT-FFR were associated with adverse outcomes. The fully integrated model combining anatomical, functional, and inflammatory CCTA-derived biomarkers improved risk stratification compared with plaque-based assessment alone. **Conclusions:** Automated multiparametric CCTA phenotyping may provide complementary prognostic information for risk stratification in patients with NOCAD. The diabetes-specific findings should be considered exploratory and require validation in larger prospective cohorts.

## Simple Summary

Patients with diabetes may still experience cardiovascular events even when coronary artery narrowing is not severe. Coronary computed tomography angiography can provide more information than narrowing alone by assessing plaque burden, plaque vulnerability, blood flow impairment, and inflammation around coronary arteries. In this study, we found that combining these imaging features improved the prediction of adverse cardiovascular events in patients with nonobstructive coronary artery disease. These findings suggest that comprehensive CCTA-derived imaging biomarkers may help radiologists and clinicians identify higher-risk patients and support more personalized follow-up and prevention strategies. However, the diabetes-specific findings should be interpreted as exploratory and require further validation.

## 1. Introduction

Diabetes mellitus (DM) markedly accelerates the development and progression of atherosclerosis and is associated with increased cardiovascular morbidity and mortality. Importantly, cardiovascular events in patients with DM frequently occur in the absence of flow-limiting epicardial coronary stenosis [1]. This phenomenon has been attributed to multiple diabetes-related pathophysiological mechanisms, including coronary microvascular dysfunction, chronic metabolic inflammation, diffuse plaque burden, and heightened plaque vulnerability [2,3]. As a result, a considerable proportion of patients with DM present with nonobstructive coronary artery disease (NOCAD) yet remain exposed to residual cardiovascular risk. In this population, conventional stenosis-based risk stratification may be insufficient for identifying individuals at increased risk of adverse events [4,5,6].

Coronary computed tomography angiography (CCTA) is currently recommended as a first-line imaging modality for the evaluation of suspected coronary artery disease. However, the limitations of luminal stenosis assessment are particularly pronounced in patients with DM. Diffuse atherosclerosis, positive vessel remodeling, and microvascular dysfunction may weaken the relationship between anatomic stenosis severity and downstream myocardial ischemia, thereby masking clinically relevant disease activity on routine CCTA interpretation. Consequently, reliance on stenosis severity alone may underestimate disease burden and prognostic risk in diabetic patients with NOCAD [7,8].

In this context, advanced CCTA-derived biomarkers, such as CT-derived fractional flow reserve (CT-FFR), quantitative plaque characteristics, and the perivascular fat attenuation index (FAI), have emerged as complementary tools that extend beyond luminal narrowing. CT-FFR provides lesion-specific functional assessment of coronary hemodynamics, quantitative plaque analysis enables objective characterization of global atherosclerotic burden and high-risk plaque phenotypes, and perivascular FAI serves as a noninvasive marker of coronary inflammation, a key driver of plaque vulnerability [9]. Prior works have demonstrated the prognostic relevance of CT-FFR in diabetes and the importance of plaque burden in NOCAD [10,11,12]. Nevertheless, most investigations to date have relied on semi-automated or operator-dependent workflows, which may limit reproducibility, scalability, and clinical implementation—particularly when multiple imaging biomarkers are evaluated concurrently.

Recent advances in artificial intelligence (AI) have enabled fully automated extraction of plaque metrics, CT-FFR, and FAI measurement from routine CCTA datasets. Such automated workflows allow standardized, operator-independent assessment of anatomical, functional, and inflammatory coronary phenotypes within a single imaging framework, offering the potential for more comprehensive and reproducible risk stratification [13,14]. However, the incremental prognostic value of integrating these automated CCTA-derived biomarkers has not been specifically evaluated in patients with NOCAD, particularly among those with DM. Accordingly, the present study aimed to investigate whether an automated CCTA-based workflow integrating quantitative plaque analysis, CT-FFR, and perivascular FAI provides incremental prognostic value beyond conventional clinical and stenosis-based assessment in patients with NOCAD, with exploratory evaluation in the diabetic subgroup.

## 2. Materials and Methods

### 2.1. Study Population

**Figure 1:**
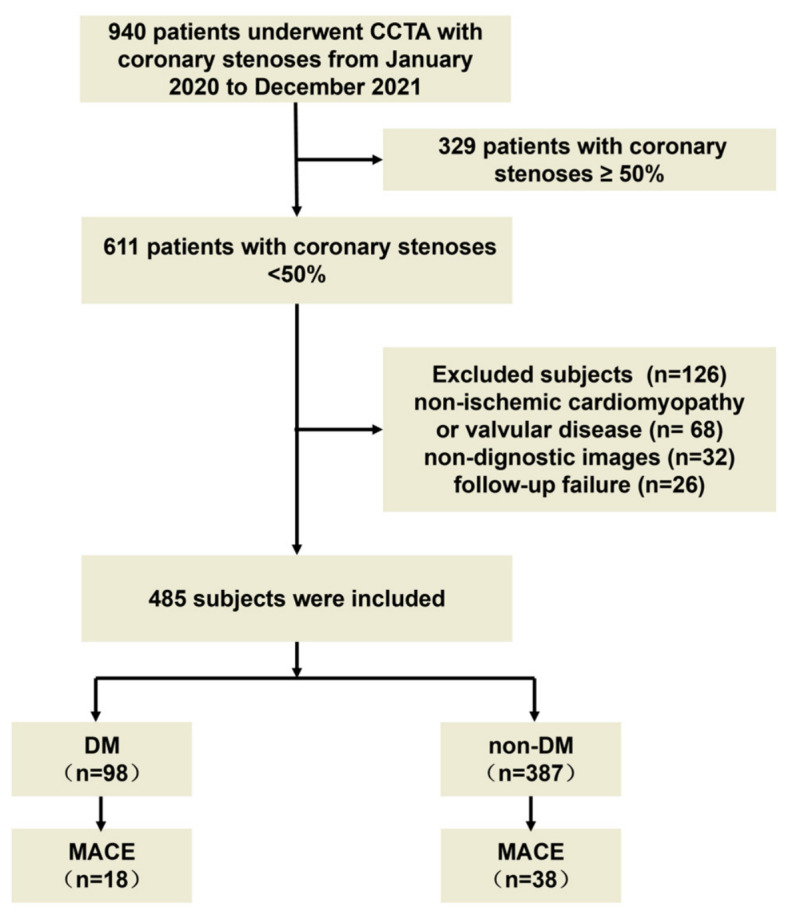
Study flowchart. CCTA = coronary computed tomography angiography; CT-FFR = CT-derived fractional flow reserve; MACE = major adverse cardiovascular event; DM = diabetes mellitus.

This retrospective study was approved by the institutional review board of our hospital (approval number: LS2025211), and the requirement for informed consent was waived. The study was conducted in accordance with the Declaration of Helsinki. Consecutive patients (>18 years) who underwent CCTA for CAD between January 2020 and December 2021 were screened for eligibility. Inclusion criteria were as follows: availability of complete baseline data, including demographic characteristics, cardiovascular risk factors, and laboratory results; adequate CCTA image quality for diagnostic evaluation; and presence of nonobstructive coronary lesion, defined as <50% diameter stenosis in all major epicardial vessels on CCTA [15]. Exclusion criteria were as follows: history of coronary revascularization or myocardial infarction; congenital or structural heart disease; anomalous coronary artery origin or termination; failure of plaque analysis, CT-FFR computation, or FAI quantification; incomplete clinical or follow-up data. DM was defined based on documented medical history or the use of glucose-lowering medications. Baseline clinical and laboratory data were retrieved from the electronic medical record system. The overall study flow and patient selection process are presented in Figure 1.

### 2.2. CCTA Scanning Protocol

All CCTA examinations were performed using a third-generation dual-source CT scanner (Somatom Definition Force, Siemens Healthineers, Forchheim, Germany). Prior to image acquisition, patients received standardized breath-hold training to minimize motion artifacts. Image acquisition was performed from 1 cm below the tracheal carina to 1–2 cm below the cardiac apex, in accordance with established CCTA guidelines. Prospective electrocardiographic (ECG) triggering was applied, typically covering 30–80% of the R-R interval. Tube voltage was set at 100–120 kV with automatic tube current modulation. Additional acquisition parameters included detector collimation of 128 × 0.6 mm, gantry rotation time of 0.25 s, pitch of 1.2, matrix size of 512 × 512, and a temporal resolution of approximately 75 ms. Images were reconstructed with a slice thickness of 1.0 mm and an iterative reconstruction algorithm. A nonionic iodinated contrast agent (iopromide 370 mg I/mL; 50–70 mL) was administered via an antecubital vein at a rate of 4–5 mL/s, followed by a 30–40 mL saline flush at the same rate. Bolus tracking was employed, and image acquisition commenced 6 s after attenuation in the ascending aorta reached 100 Hounsfield units. The cardiac phase with optimal image quality was selected for post-processing analysis.

### 2.3. CCTA Image Analysis

All CCTA datasets were analyzed using a dedicated workstation for quantitative coronary evaluation (CoronaryDoc, version 1.0.4; Shukun Technology, Beijing, China). The software automatically segmented the entire coronary tree and generated multiplanar reformations, cross-sectional images, and maximum intensity projections. It also provided automated plaque segmentation and quantitative outputs. All automatically generated coronary centerlines, lumen contours, plaque segmentations, and quantitative results were independently reviewed by two radiologists, L.C. and H.H., with 6 and 8 years of experience in cardiovascular CT imaging, respectively. Both readers were blinded to clinical outcomes. Manual adjustment was performed when necessary to ensure accurate lumen and vessel boundary definition, and discrepancies were resolved by consensus. To comprehensively characterize coronary burden, plaque measurements were conducted at both the lesion and the patient levels. For each target lesion, quantitative parameters, including percentage diameter stenosis (%DS), plaque length, minimum lumen area (MLA), total plaque volume (TPV), and volumes of calcified and noncalcified components, were derived using standardized CT plaque quantification protocols. At the patient level, total plaque burden (PB) and TPV were calculated by summing plaque metrics across all major epicardial vessels, providing a global assessment of coronary atherosclerotic burden. PB was calculated as the ratio of plaque volume to vessel volume [16]. Plaque composition was classified according to the predefined voxel-based CT attenuation thresholds of the software, including lipid-rich plaque (−100 to 30 HU), fibrous plaque (30 to 150 HU), and calcified plaque (150 to 350 HU). Noncalcified PV was defined as the sum of lipid-rich and fibrous plaque volumes. FAI was automatically quantified by delineating pericoronary adipose tissue with attenuation values between −190 and −30 HU surrounding the major coronary arteries [17]. In the present automated analysis framework, patient-specific FAI was calculated as the mean attenuation within a 3 mm radial perivascular layer along the longitudinal course of the corresponding plaque, reflecting plaque-associated perivascular adipose tissue attenuation. This automated analysis pipeline has been validated in prior studies and enables standardized, operator-independent quantification of coronary imaging biomarkers.

### 2.4. CT-FFR Analysis

CT-FFR was computed using a fully automated, on-site software package (skCT-FFR, version 0.7.1; Shukun Technology, Beijing, China), approved by the China National Medical Products Administration [18,19]. After importing routine CCTA datasets, the software automatically generated coronary centerlines and lumen contours; manual correction was performed when necessary to ensure optimal segmentation quality. The algorithm integrates a reduced-order fluid dynamics framework with machine-learning techniques, including geometric feature compression, flow-field decomposition, and nonlinear regression trained on large anatomic hemodynamic datasets. This approach enables rapid, patient-specific FFR computation without the need for time-intensive full CFD simulations. For each lesion, CT-FFR values were measured 2–4 cm distal to the site of maximal luminal narrowing. In patients with more than one stenotic vessel, the lowest CT-FFR value across the entire coronary tree was recorded as the patient-level CT-FFR index. This patient-level approach was used to reflect global functional impairment and was not intended to identify the culprit lesion responsible for subsequent events.

### 2.5. Clinical Outcomes

Clinical follow-up was obtained through systematic review of electronic medical records, outpatient clinic visits, and structured telephone interviews. The primary endpoint was the occurrence of MACE, defined as a composite of all-cause death, nonfatal myocardial infarction, urgent coronary revascularization during follow-up, and hospitalization for unstable angina. Events were adjudicated at the patient level. Therefore, nonfatal myocardial infarction was not required to occur in the vessel with the lowest CT-FFR value. All events were adjudicated by a dedicated clinical events committee according to standardized definitions [20].

### 2.6. Statistical Analysis

Continuous variables were tested for normality using the one-sample Kolmogorov–Smirnov test. Normally distributed variables are expressed as mean ± standard deviation and compared using the independent-sample Student’s t-test. Non-normally distributed variables are presented as median (interquartile range) and were compared using the Mann–Whitney U test. Categorical variables are summarized as counts and percentages and were compared using the chi-square test or Fisher’s exact test, as appropriate. Inter-observer agreement for quantitative CCTA parameters was evaluated using the intraclass correlation coefficient (ICC), whereas agreement for qualitative plaque characteristics was assessed with Cohen’s κ statistic. Event-free survival was estimated using Kaplan–Meier curves and compared with the log-rank test. Associations between CCTA-derived plaque characteristics, FAI, CT-FFR, and MACE were assessed using univariate Cox proportional hazards regression. Variables with *p* ≤ 0.05 in univariate analysis were entered into multivariate Cox regression models, with results reported as hazard ratios (HRs) and 95% confidence intervals (CIs). To evaluate the incremental prognostic value of CCTA-derived biomarkers, hierarchical Cox proportional hazards models were constructed. Model discrimination was assessed using Harrell’s concordance index (C-index). Internal validation was performed using bootstrap resampling (1000 repetitions) to estimate optimism-corrected C-indices and calibration slopes. Incremental prognostic performance was further assessed using net reclassification improvement (NRI) and integrated discrimination improvement (IDI). Events-per-variable (EPV) ratios were calculated to describe model complexity. Exploratory interaction analyses were conducted by introducing diabetes-by-biomarker interaction terms into multivariable Cox models. All statistical tests were two-tailed, and a *p* < 0.05 was considered statistically significant. Statistical analyses were performed using SPSS (version 26.0, IBM Corp., Armonk, NY, USA), MedCalc (version 19.0, Ostend, Belgium), and R software (version 4.3.3; R Foundation for Statistical Computing, Vienna, Austria).

## 3. Results

### 3.1. Baseline Clinical Characteristics

Between January 2020 and December 2021, a total of 940 consecutive patients undergoing CCTA were screened for eligibility. After exclusion of patients who did not meet the inclusion criteria or had incomplete imaging or follow-up data, 485 patients were finally included in the analysis (Figure 1). These included 98 patients with DM (mean age 66.8 ± 7.1 years, 45.9% male) and 387 patients without DM (mean age 66.4 ± 8.4 years, 43.2% male). As expected, fasting plasma glucose levels were significantly higher in patients with DM compared with those without DM (median 6.2 vs. 5.3 mmol/L, *p* < 0.001). No other baseline parameters differed significantly between the two groups (all *p* > 0.05, Table 1). During a median follow-up of 36 months, MACE occurred in 56 patients (11.5%). The incidence of MACE was significantly higher in the DM group than in the non-DM group (18.4% vs. 9.8%, *p* = 0.018), primarily driven by a higher rate of hospitalization for unstable angina (10.2% vs. 4.9%, *p* = 0.048). No significant between-group differences were observed for all-cause death, nonfatal myocardial infarction, or urgent coronary revascularization.

### 3.2. CCTA Findings

**Figure 2:**
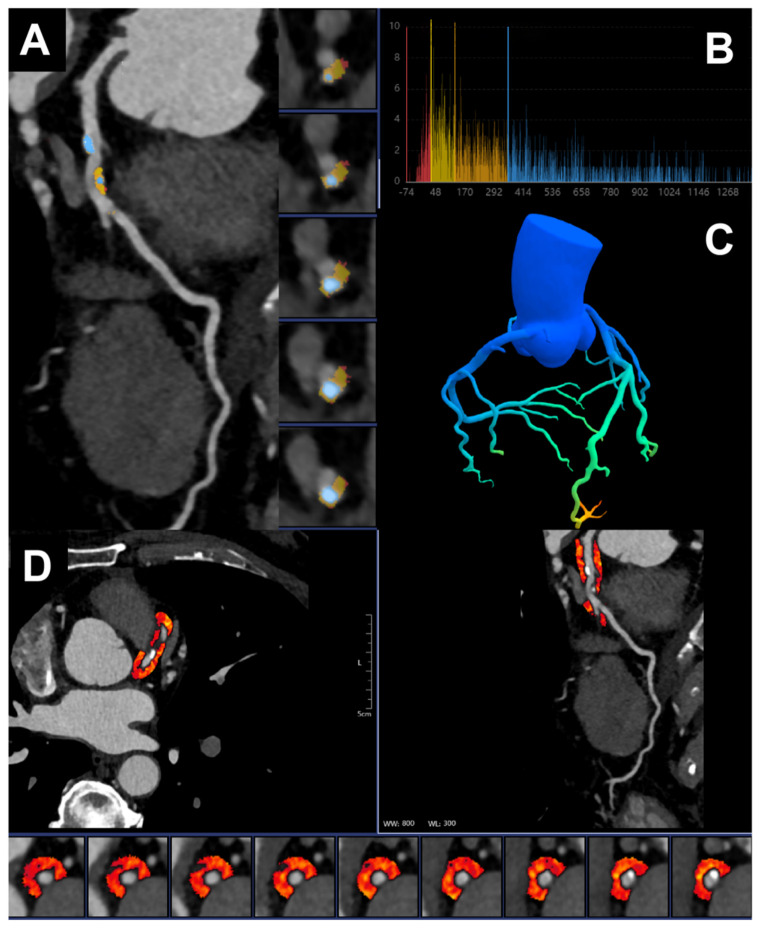
Representative multiparametric CCTA findings in a patient with DM. Curved planar reconstruction demonstrated mixed plaque in the proximal left anterior descending artery (**A**). Quantitative plaque analysis (**B**). CT-FFR value of 0.86 (**C**). Perivascular FAI of −69.2 HU (**D**). This patient experienced MACE during follow-up.

Inter- and intra-observer reproducibility for quantitative plaque characteristics, FAI, and CT-FFR was excellent, with ICC values ranging from 0.78 to 0.93. Comparisons of CCTA-derived plaque characteristics between patients with and without DM are presented in Table 2. Patients with DM showed a significantly higher overall atherosclerotic burden, characterized by greater PV (236.3 vs. 205.8 mm^3^, *p* = 0.027), higher noncalcified PV (143.1 vs. 115.7 mm^3^, *p* = 0.019), and increased PB (48.9% vs. 42.7%, *p* = 0.025). In addition, diabetic patients exhibited higher FAI values (−71.5 HU vs. −79.2 HU, *p* = 0.011) and lower CT-FFR values (0.86 vs. 0.90, *p* = 0.013), reflecting more pronounced functional impairment. HRP features were significantly more prevalent in the DM group than in the non-DM group (10.2% vs. 3.4%, *p* = 0.004). Other CCTA-derived parameters, including plaque length and minimum lumen area, did not differ significantly between groups. A representative diabetic patient with NOCAD who demonstrated mixed plaque, reduced CT-FFR, elevated perivascular FAI, and subsequent MACE during follow-up is shown in Figure 2.

### 3.3. Association of CCTA Measures with MACE According to Diabetes Status

**Figure 3:**
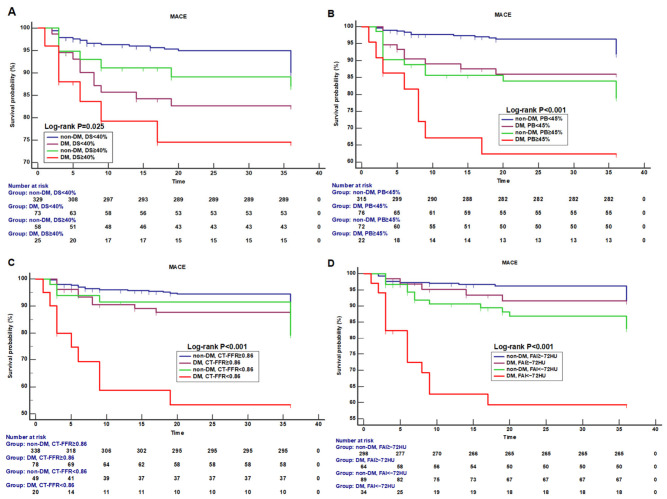
Kaplan–Meier survival curves stratified by imaging biomarkers. Kaplan–Meier curves for MACE stratified by DS (**A**), PB (**B**), CT-FFR (**C**), and FAI (**D**).

ROC analysis was performed to identify optimal cutoff values for key imaging parameters associated with MACE. The optimal thresholds were 40% for percentage diameter stenosis (%DS), 45% for total PB, 0.86 for CT-FFR, and −72 HU for FAI. Kaplan–Meier survival analysis based on these thresholds demonstrated consistent and graded separation of event-free survival according to CCTA-derived imaging biomarkers, with more pronounced risk stratification observed in patients with DM compared with those without DM (Figure 3). Stratification by percentage diameter stenosis (%DS ≥ 40%) showed significantly lower event-free survival (log-rank *p* = 0.025), with diabetic patients exhibiting earlier and steeper declines in survival probability. Total PB (≥45%) provided robust risk stratification, and patients with high plaque burden experienced markedly increased MACE rates (log-rank *p* < 0.001), particularly in the DM group. Reduced CT-FFR (≤0.86) was also associated with a significantly higher risk of MACE (log-rank *p* < 0.001), with earlier curve separation observed in diabetic patients. Similarly, elevated FAI (>−72 HU) demonstrated significant prognostic discrimination (log-rank *p* < 0.001), with rapid early event accumulation in patients with DM.

In multivariate Cox regression analyses stratified by DM status, distinct prognostic patterns were observed (Table 3). Among patients with DM, total PB ≥ 45% (HR: 4.642, 95% CI: 1.158–18.606; *p* = 0.030), presence of HRP (HR: 5.496, 95% CI: 1.039–29.087; *p* = 0.045), and elevated FAI (HR: 4.659, 95% CI: 1.181–18.383; *p* = 0.028) remained associated with MACE after multivariable adjustment. CT-FFR ≤ 0.86 showed a borderline association with adverse outcomes in patients with DM (HR: 4.180, 95% CI: 0.982–17.782; *p* = 0.053). In the non-DM group, total PB ≥ 45%, HRP, and elevated FAI also remained independently associated with MACE, albeit with comparatively lower hazard ratios, while CT-FFR did not retain independent prognostic significance. No statistically significant diabetes-by-biomarker interactions were observed, although trends were noted for total plaque burden and FAI (*p* for interaction 0.061–0.073).

### 3.4. Incremental Prognostic Value of Integrated CCTA Models

To assess the incremental prognostic value of integrated CCTA-derived biomarkers, four hierarchical Cox regression models were constructed in the overall study cohort (Table 4). Model 1 included total PB and HRP features. Model 2 incorporated CT-FFR into Model 1. Model 3 added FAI, and Model 4 represented the fully integrated model combining PB, HRP, CT-FFR, and FAI. Model 1 demonstrated modest predictive performance (C-index = 0.662; 95% CI: 0.580–0.740). Discrimination increased with the addition of CT-FFR (Model 2: C-index = 0.734; 95% CI: 0.652–0.816) and FAI (Model 3: C-index = 0.756; 95% CI: 0.673–0.838). The fully integrated Model 4 demonstrated the highest apparent C-index (0.829), with an optimism-corrected C-index of 0.777 after bootstrap validation.

In bootstrap internal validation with 1000 repetitions, estimated optimism increased with model complexity (0.021 for Model 1, 0.031 for Model 2, 0.038 for Model 3, and 0.052 for Model 4). After optimism correction, the C-indices were 0.641, 0.703, 0.718, and 0.777 for Models 1–4, respectively. The corrected calibration slope for Model 4 was 0.88, indicating acceptable model stability with mild overfitting. Compared with Model 1, the fully integrated Model 4 significantly improved risk reclassification. The apparent NRI was 0.368, and the integrated discrimination improvement (IDI) was 0.174. After bootstrap correction, NRI and IDI remained significant (corrected NRI = 0.318; corrected IDI = 0.142).

In the diabetic subgroup, discrimination similarly improved with the addition of functional and inflammatory biomarkers. ROC analysis demonstrated higher AUCs for integrated models compared with the plaque-only model, and DeLong tests confirmed statistically significant improvements (all *p* < 0.01), supporting the incremental prognostic value of multiparametric CCTA phenotyping in patients with diabetes mellitus. However, only 18 MACEs occurred in the DM subgroup. Accordingly, the EPV values were limited, particularly for the fully integrated four-predictor model, which yielded an EPV of approximately 4.5. Therefore, the diabetes-specific Cox regression results should be interpreted cautiously.

## 4. Discussion

In this study, we investigated the prognostic implications of multiparametric CCTA-derived imaging biomarkers in patients with NOCAD, with exploratory assessment in individuals with DM. Over a median follow-up of three years, MACE occurred in 11.5% of the overall cohort and was more frequent in patients with DM than in those without DM. Our principal findings are threefold. First, patients with DM showed a greater overall atherosclerotic burden, higher noncalcified plaque volume, more frequent high-risk plaque features, higher FAI values, and lower CT-FFR values, despite the absence of obstructive coronary stenosis. Second, plaque burden, high-risk plaque features, FAI, and CT-FFR were associated with adverse outcomes, although their relative prognostic contribution differed according to diabetes status. Third, the integrated model combining structural, functional, and inflammatory CCTA-derived biomarkers improved risk discrimination compared with plaque-based assessment alone. However, diabetes-by-biomarker interaction testing did not reach statistical significance, and the diabetes-specific analyses were limited by the small number of events. Therefore, these subgroup findings should be considered exploratory and hypothesis-generating rather than confirmatory.

DM is characterized by diffuse atherosclerosis, chronic low-grade inflammation, endothelial dysfunction, and early microvascular dysfunction, which together confer excess cardiovascular risk even in the absence of flow-limiting coronary stenosis [21]. Consistent with this pathophysiological background, our study showed that patients with DM had a higher incidence of MACE despite nonobstructive coronary lesions [4,22,23]. These findings support the concept that stenosis severity alone may be insufficient for risk stratification in patients with DM and NOCAD. A comprehensive assessment of plaque burden, plaque vulnerability, vascular inflammation, and functional impairment may provide additional information beyond luminal narrowing.

Functional assessment with CT-FFR provided additional prognostic information; however, its incremental value was modest when applied in isolation to diabetic NOCAD patients. Notably, the optimal CT-FFR threshold associated with MACE in our study was 0.86, higher than the conventional ischemic cutoff of 0.80 [24]. This finding may reflect pathophysiological features of diabetes, in which microvascular dysfunction and impaired vasodilatory reserve attenuate translesional pressure gradients, thereby limiting the discriminatory capacity of CT-FFR in nonobstructive disease [25]. Consequently, in diabetic patients who often exhibit preserved epicardial flow but impaired microcirculation, functional assessment alone may fail to fully capture residual cardiovascular risk. This internally derived threshold should be interpreted as an exploratory risk-stratification cutoff rather than a new clinical decision threshold.

In contrast, imaging markers that reflect plaque biology and vascular inflammation appear particularly informative in diabetes. FAI serves as a surrogate marker of coronary inflammation, a central driver of plaque destabilization in diabetic atherosclerosis. Prior studies have shown that diabetes is associated with heightened inflammatory activity within both the vascular wall and surrounding adipose tissue, potentially increasing the risk of plaque progression and rupture [26,27,28]. Consistent with this mechanism, we observed that elevated FAI remained associated with MACE after adjustment, with an optimal prognostic threshold of −72 HU. These findings suggest that FAI captures a biologically active disease process that is especially relevant in diabetic patients, beyond what can be inferred from stenosis severity or ischemia alone.

Similarly, quantitative plaque analysis revealed that diabetic patients exhibited greater plaque burden and a higher prevalence of HRP features. HRPs characterized by large necrotic cores, positive remodeling, and thin fibrous caps are particularly susceptible to rupture under conditions of inflammation and altered shear stress, both of which are exacerbated in diabetes [29,30]. In our study, HRP demonstrated a strong association with MACE, exceeding that of CT-FFR. This observation underscores the concept that, in diabetic NOCAD, plaque vulnerability rather than flow-limiting stenosis may be an important determinant of adverse outcomes.

The fully integrated model combining plaque characteristics, CT-FFR, and FAI demonstrated the highest apparent discrimination and maintained acceptable performance after bootstrap correction. This finding is biologically plausible because these biomarkers represent complementary domains of coronary disease: structural atherosclerotic burden, plaque vulnerability, functional impairment, and perivascular inflammation [9,31,32]. The optimism-corrected C-index of the fully integrated model remained higher than that of the plaque-based model, suggesting that multiparametric CCTA phenotyping may improve risk stratification in patients with NOCAD. Nevertheless, the improvement should be interpreted in the context of internal validation only. The calibration slope indicated mild overfitting, and the imaging cutoffs were derived from the same cohort. External validation is therefore required before this model can be generalized or used for clinical decision-making.

From a clinical perspective, the present findings suggest that patients with NOCAD, particularly those with DM, should not be considered uniformly low risk based solely on the absence of obstructive stenosis [4,5,6,22,23]. Automated multiparametric CCTA analysis may help identify patients with more adverse coronary phenotypes who may benefit from closer surveillance and more intensive optimization of guideline-directed preventive therapy, including lipid-lowering therapy, glycemic control, blood pressure management, and lifestyle intervention. Importantly, these findings should not be interpreted as supporting prophylactic revascularization for nonobstructive lesions in the absence of severe stenosis or clear physiological significance. The potential clinical value of this approach lies in improved risk stratification and individualized prevention. Whether imaging-guided intensification of preventive therapy can improve clinical outcomes requires prospective evaluation.

Several limitations should be acknowledged. First, this was a single-center retrospective study without external validation, and residual optimism may remain despite internal validation. The exclusion of patients because of ineligibility, inadequate image quality, failed post-processing, or incomplete follow-up may also have introduced selection bias. Second, the diabetes-specific analyses were limited by the small subgroup size. Only 98 patients had diabetes mellitus, with 18 MACEs, resulting in limited statistical power and a low events-per-variable ratio for multivariable modeling. Therefore, these subgroup findings should be considered exploratory and hypothesis-generating. Third, several relevant clinical variables were unavailable, including HbA1c, diabetes duration, antidiabetic drug classes, insulin use, and detailed statin treatment information. These unmeasured factors may have influenced plaque characteristics, perivascular inflammation, FAI values, and clinical outcomes, leading to potential residual confounding. Fourth, the imaging cutoffs, including the CT-FFR threshold of 0.86, were internally derived and not externally validated. Given the limited number of patients with CT-FFR ≤ 0.80 in this nonobstructive CAD cohort, the 0.86 threshold should be interpreted as an exploratory cohort-specific cutoff rather than a universal clinical decision threshold. Fifth, although standardized CCTA acquisition and automated analysis were used, plaque metrics and FAI measurements may still be affected by image quality, calcification, motion artifacts, and residual technical variability. In addition, CT-FFR and MACE were analyzed at the patient level rather than the vessel or lesion level, preventing direct linkage between specific imaging abnormalities and subsequent events. Finally, the composite endpoint included hospitalization for unstable angina, which may be influenced by clinical decision-making in a retrospective, non-blinded design. The limited number of hard endpoint events precluded sensitivity analyses restricted to death, myocardial infarction, or revascularization. Larger prospective multicenter studies with external validation and lesion-level analyses are needed.

## 5. Conclusions

In conclusion, automated multiparametric CCTA phenotyping may provide complementary prognostic information for risk stratification in patients with NOCAD. The integration of CT-FFR, quantitative plaque characteristics, and perivascular FAI may help identify patients with higher cardiovascular risk beyond stenosis severity alone. The diabetes-specific findings should be considered exploratory and hypothesis-generating. Further prospective multicenter studies are needed to validate these findings and determine whether imaging-guided risk stratification can support more individualized preventive management.

## Figures and Tables

**Table 1:** Patients’ baseline characteristics.

Variables	DM Group (n = 98)	Non-DM Group (n = 387)	*p* Value
**Baseline characteristics**			
Age, yrs	66.8 ± 7.11	66.4 ± 8.1	0.602
Sex (male), n (%)	45 (45.9)	167 (43.2)	0.622
BMI, kg/m^2^	24.8 ± 2.8	24.7 ± 2.6	0.710
**Cardiac risk factors**			
Hypertension, n (%)	58 (59.2)	195 (50.4)	0.119
Hyperlipidemia, n (%)	38 (38.8)	129 (33.3)	0.311
Smoking, n (%)	33 (33.7)	99 (25.6)	0.108
Family history of CAD, n (%)	29 (29.6)	73 (23.1)	0.174
**Medication**			
Aspirin	39 (39.8)	147 (38.0)	0.742
Statin	35 (35.4)	124 (32.2)	0.552
Beta blocker	22 (22.4)	75 (19.4)	0.497
ACEi/ARB	17 (17.4)	61 (15.8)	0.718
**Symptom**			0.462
No chest pain	41 (41.8)	135 (34.9)	
Noncardiac chest pain	12 (12.2)	69 (17.8)	
Typical chest pain	13 (13.3)	50 (12.9)	
Atypical chest pain	32 (32.7)	133 (34.4)	
**Laboratory results**			
Creatinine, µmol/L	71.5 ± 20.7	72.3 ± 18.7	0.281
eGFR, mL/min/1.73 m^2^	76.3 ± 25.6	82.4 ± 23.0	0.198
Fasting glucose, mmol/L	6.2 (5.1, 6.6)	5.3 (5.0, 5.9)	<0.001
Total cholesterol, mmol/L	4.61 (3.42, 5.69)	4.53 (3.54, 5.38)	0.497
LDL cholesterol, mmol/L	2.96 (2.12, 3.72)	2.91 (2.01, 3.83)	0.646
HDL cholesterol, mmol/L	1.08 ± 0.27	1.05 ± 0.32	0.724
Triglycerides, mmol/L	1.46 (0.90, 2.17)	1.31 (1.01, 2.09)	0.364
**MACEs, n (%)**	18 (18.4)	38 (9.8)	0.018
All-cause death, n (%)	2 (2.0)	5 (1.3)	0.633
Non-fatal myocardial infarction, n (%)	2 (2.0)	6 (1.6)	0.666
Urgent coronary revascularization, n (%)	4 (4.1)	8 (2.1)	0.210
Hospitalization for unstable angina, n (%)	10 (10.2)	18 (4.9)	0.048

Values are mean ± standard deviation, n (%), or median (interquartile range). DM = diabetes mellitus; ACEi = angiotensin-converting enzyme inhibitor; ARB = angiotensin II receptor blocker; CAD = coronary artery disease; eGFR = estimated glomerular filtration rate; HDL = high-density lipoprotein; LDL = low-density lipoprotein; MACEs = major adverse cardiovascular events.

**Table 2:** Comparison of CCTA plaque characteristics, FAI, and CT-FFR.

Variables	DM Group (n = 98)	Non-DM Group (n = 387)	*p* Value
%DS	37.4 ± 7.2	35.1 ± 7.6	0.007
Plaque length (mm)	27.9 ± 8.6	26.8 ± 5.7	0.129
MLA (mm^2^)	5.5 [4.0, 7.2]	6.1 [4.8, 7.8]	0.134
PV (mm^3^)	236.3 [151.3, 357.9]	205.8 [136.0, 304.9]	0.027
Calcified PV (mm^3^)	77.7 [54.9, 147.3]	81.8 [45.7, 122.2]	0.150
Noncalcified PV (mm^3^)	143.1 [86.2, 191.3]	115.7 [75.8, 167.2]	0.019
Total PB (%)	48.9 ± 5.0	42.7 ± 4.1	0.025
Calcified PB (%)	16.4 ± 2.6	15.0 ± 2.2	0.564
Noncalcified PB (%)	32.9 ± 4.7	28.7 ± 4.8	0.053
FAI (HU)	−71.5 (−79.3, −65.6)	−79.2 (−85.5, −70.9)	0.011
CT-FFR	0.86 (0.81, 0.94)	0.90 (0.85, 0.96)	0.013
HRP, n (%)	10 (10.2)	13 (3.4)	0.004

%DS = percentage diameter stenosis; MLA = minimum lumen area; PV = plaque volume; PB = plaque burden; FAI = fat attenuation index; CT-FFR = fractional flow reserve derived from computed tomography; HRP = high-risk plaque.

**Table 3:** Multivariable Cox proportional hazards regression for predictors of MACE.

Variables	Univariate Analysis			Multivariate Analysis		
	HR	95% CI	*p* Value	HR	95% CI	*p* Value
**Patients with DM**						
%DS	1.605	0.531–4.857	0.402	1.029	0.240–4.418	0.969
PV	1.000	0.996–1.003	0.876	1.003	0.994–1.012	0.501
Noncalcified PV	0.998	0.992–1.004	0.431	0.993	0.979–1.007	0.336
Total PB	3.771	1.263–11.263	0.017	4.642	1.158–18.606	0.030
HRP	9.500	2.333–38.680	0.002	5.496	1.039–29.087	0.045
FAI	7.305	2.324–22.963	0.006	4.659	1.181–18.383	0.028
CT-FFR	6.273	2.043–19.261	0.001	4.180	0.982–17.782	0.053
**Patients with non-DM**						
%DS	1.319	0.552–3.157	0.225	0.934	0.367–2.378	0.887
PV	1.004	1.001–1.007	0.002	1.002	0.998–1.007	0.313
Noncalcified PV	1.006	1.002–1.009	0.002	1.002	0.995–1.009	0.567
Total PB	2.927	1.429–5.994	0.003	2.304	1.034–5.133	0.041
HRP	4.255	1.412–12.819	0.010	4.074	1.276–13.011	0.018
FAI	2.131	1.051–4.321	0.036	2.155	1.015–4.573	0.046
CT-FFR	2.397	1.059–5.428	0.036	2.288	0.957–5.715	0.063

HR = hazard ratio; CI = confidence interval; other abbreviations are defined in Table 2.

**Table 4:** Model complexity and internal validation of Cox regression models for predicting MACE.

Model	Model 1 (PB + HRP)	Model 2 (Model 1 + CT-FFR)	Model 3 (Model 1 + FAI)	Model 4 (Model 1 + CT-FFR + FAI)
Predictors (n)	2	3	3	4
Events in overall cohort (n)	56	56	56	56
EPV in overall cohort	28.0	18.7	18.7	14.0
Events in diabetic subgroup (n)	18	18	18	18
EPV in diabetic subgroup	9.0	6.0	6.0	4.5
C-index (apparent)	0.662	0.734	0.756	0.829
Bootstrap 95% CI (C-index)	0.580–0.740	0.652–0.816	0.673–0.838	0.755–0.897
Estimated optimism (C-index) *	0.021	0.031	0.038	0.052
Estimated optimism-corrected C-index *	0.641	0.703	0.718	0.777
Incremental comparison	**Reference**	**vs. Model 1**	**vs. Model 1**	**vs. Model 1**
NRI	NA	0.212	0.245	0.368
Bootstrap 95% CI (NRI)	NA	0.041–0.383	0.068–0.422	0.112–0.624
Estimated optimism-corrected NRI *	NA	0.185	0.214	0.318
IDI	NA	0.098	0.112	0.174
Bootstrap 95% CI (IDI)	NA	0.028–0.168	0.036–0.188	0.059–0.289
Estimated optimism-corrected IDI *	NA	0.081	0.094	0.142

EPV = events per variable; NRI = net reclassification improvement; IDI = integrated discrimination improvement; other abbreviations are defined in Table 2 and Table 3. * Optimism was estimated using bootstrap resampling with 1000 repetitions, and optimism-corrected discrimination and reclassification metrics were calculated based on the average bootstrap optimism.

## Data Availability

The data that support the findings of this study are available from the corresponding author upon reasonable request due to privacy and institutional restrictions.

## Abbreviations

The following abbreviations are used in this manuscript:

BMI	Body mass index
CAD	Coronary artery disease
CCTA	Coronary computed tomography angiography
CI	Confidence interval
CT-FFR	CT-derived fractional flow reserve
DM	Diabetes mellitus
%DS	Percentage diameter stenosis
FAI	Fat attenuation index
HR	Hazard ratio
HRP	High-risk plaque
ICC	Intraclass correlation coefficient
IDI	Integrated discrimination improvement
KM	Kaplan–Meier
MACE	Major adverse cardiovascular events
NOCAD	Nonobstructive coronary artery disease
NRI	Net reclassification improvement
PV	Plaque volume
PB	Plaque burden
ROC	Receiver operating characteristic

## References

[1] Karakasis P., Theofilis P., Patoulias D., Vlachakis P.K., Antoniadis A.P., Fragakis N. (2025). Diabetes-driven atherosclerosis: Updated mechanistic insights and novel therapeutic strategies. Int. J. Mol. Sci..

[2] Yu Y., Yang W., Dai X., Yu L., Lan Z., Ding X., Zhang J. (2023). Microvascular myocardial ischemia in patients with diabetes without obstructive coronary stenosis and its association with angina. Korean J. Radiol..

[3] Salvatore T., Galiero R., Caturano A., Vetrano E., Loffredo G., Rinaldi L., Catalini C., Gjeloshi K., Albanese G., Di Martino A. (2022). Coronary microvascular dysfunction in diabetes mellitus: Pathogenetic mechanisms and potential therapeutic options. Biomedicines.

[4] Lee Y.A., Song S.W., Kim S.H., Jung J., Jang W.Y., Moon D., Her S.H., Yoo K.D., Moon K.W., Lee S.N. (2025). Impact of diabetes duration on major adverse cardiac events in patients with non-obstructive coronary artery disease. J. Clin. Med..

[5] Liu Z., Ding Y., Dou G., Wang X., Shan D., He B., Jing J., Li T., Chen Y., Yang J. (2023). Global trans-lesional computed tomography-derived fractional flow reserve gradient is associated with clinical outcomes in diabetic patients with non-obstructive coronary artery disease. Cardiovasc. Diabetol..

[6] Taron J., Foldyna B., Mayrhofer T., Osborne M.T., Meyersohn N., Bittner D.O., Puchner S.B., Emami H., Lu M.T., Ferencik M. (2021). Risk stratification with the use of coronary computed tomographic angiography in patients with nonobstructive coronary artery disease. JACC Cardiovasc. Imaging.

[7] Huang Q., Liu W., Sun L., Liu L., Zhang S., Yang Y., Zhu X., Liu Z. (2025). Duration of diabetes, glycemic control and intracranial atherosclerotic plaque vulnerability. Eur. J. Radiol..

[8] Kamperidis V., de Graaf M.A., Uusitalo V., Saraste A., Kuneman J.H., van den Hoogen I.J., Knuuti J., Bax J.J. (2022). Atherosclerotic plaque characteristics on quantitative coronary computed tomography angiography associated with ischemia on positron emission tomography in diabetic patients. Int. J. Cardiovasc. Imaging.

[9] Wang C., Wu X., Liu Y., Qiu X., Su H., Wang Z., Gao J. (2025). Association between coronary plaque vulnerability features and multiparametric pericoronary fat indices on coronary computed tomography angiography: A cross-sectional study. Quant. Imaging Med. Surg..

[10] Lan Z., Ding X., Yu Y., Yu L., Yang W., Dai X., Ling R., Wang Y., Yang W., Zhang J. (2023). CT-derived fractional flow reserve for prediction of major adverse cardiovascular events in diabetic patients. Cardiovasc. Diabetol..

[11] Tesche C., Baquet M., Bauer M.J., Straube F., Hartl S., Leonard T., Jochheim D., Fink D., Brandt V., Baumann S. (2023). Prognostic utility of coronary computed tomography angiography-derived plaque information on long-term outcome in patients with and without diabetes mellitus. J. Thorac. Imaging.

[12] Min J.K., Chang H.J., Andreini D., Pontone G., Guglielmo M., Bax J.J., Knaapen P., Raman S.V., Chazal R.A., Freeman A.M. (2022). Coronary CTA plaque volume severity stages according to invasive coronary angiography and fractional flow reserve. J. Cardiovasc. Comput. Tomogr..

[13] Narula J., Stuckey T.D., Nakazawa G., Ahmadi A., Matsumura M., Petersen K., Mirza S., Ng N., Mullen S., Schaap M. (2024). Prospective deep learning-based quantitative assessment of coronary plaque by computed tomography angiography compared with intravascular ultrasound: The REVEALPLAQUE study. Eur. Heart J. Cardiovasc. Imaging.

[14] Xi Y., Xu Y., Shu Z. (2024). Impact of hypertension on coronary artery plaques and FFR-CT in type 2 diabetes mellitus patients: Evaluation utilizing artificial intelligence processed coronary computed tomography angiography. Front. Artif. Intell..

[15] van Rosendael A.R., Bax A.M., Smit J.M., van den Hoogen I.J., Ma X., Al’Aref S., Achenbach S., Al-Mallah M.H., Andreini D., Berman D.S. (2020). Clinical risk factors and atherosclerotic plaque extent to define risk for major events in patients without obstructive coronary artery disease: The long-term CONFIRM registry. Eur. Heart J. Cardiovasc. Imaging.

[16] Li Y., Yao W., Wang T., Yang Q., Song K., Zhang F., Wang F., Dang Y. (2024). Association of semaglutide treatment with coronary artery inflammation in type 2 diabetes mellitus patients: A retrospective study based on pericoronary adipose tissue attenuation. Cardiovasc. Diabetol..

[17] Liu M., Zhen Y., Shang J., Dang Y., Zhang Q., Ni W., Qiao Y., Hou Y. (2024). Predictive value of lesion-specific pericoronary fat attenuation index for major adverse cardiovascular events in patients with type 2 diabetes. Cardiovasc. Diabetol..

[18] Guo B., Jiang M., Guo X., Tang C., Zhong J., Lu M., Liu C., Zhang X., Qiao H., Zhou F. (2024). Diagnostic and prognostic performance of artificial intelligence-based fully automated on-site CT-derived fractional flow reserve in patients with coronary artery disease. Sci. Bull..

[19] Guo B., Xing W., Hu C., Zha Y., Yin X., He Y., Hu S., Shi Y., Lv F., Wang R. (2024). Clinical effectiveness of automated coronary CT-derived fractional flow reserve: A Chinese randomized controlled trial. Radiology.

[20] Hicks K.A., Tcheng J.E., Bozkurt B., Chaitman B.R., Cutlip D.E., Farb A., Fonarow G.C., Jacobs J.P., Jaff M.R., Lichtman J.H. (2015). 2014 ACC/AHA key data elements and definitions for cardiovascular endpoint events in clinical trials. Circulation.

[21] Juricic S., Klac J., Stojkovic S., Tesic M., Jovanovic I., Aleksandric S., Dobric M., Zivkovic S., Maricic B., Simeunovic D. (2025). Molecular and pathophysiological mechanisms leading to ischemic heart disease in patients with diabetes mellitus. Int. J. Mol. Sci..

[22] Kreimer F., Schlettert C., Abumayyaleh M., Akin I., Hijazi M.M., Hamdani N., Gotzmann M., Mügge A., El-Battrawy I., Aweimer A. (2024). Impact of diabetes mellitus on the outcome of troponin-positive patients with non-obstructive coronary arteries. Int. J. Cardiol. Heart Vasc..

[23] Lai C.C., Chang B.C., Hwang L.C. (2025). Presence of coronary artery disease in adults with newly detected diabetes mellitus. BMC Cardiovasc. Disord..

[24] Zhao Q., Liu L., Xian H., Luo X., Zhang D., Hou S., Qu C., Zhang R., Qu X. (2024). Prognostic value of computed tomography-derived fractional flow reserve in patients with diabetes mellitus and unstable angina. Cardiovasc. Diabetol..

[25] Gallinoro E., Paolisso P., Candreva A., Bermpeis K., Fabbricatore D., Esposito G., Bertolone D., Fernandez Peregrina E., Munhoz D., Mileva N. (2021). Microvascular dysfunction in patients with type II diabetes mellitus: Invasive assessment of absolute coronary blood flow and microvascular resistance reserve. Front. Cardiovasc. Med..

[26] Overgaard K.S., Andersen T.R., Heinsen L.J., Pararajasingam G., Mohamed R.A., Madsen F.S., Biesenbach I.I.A., Højlund K., Lambrechtsen J., Auscher S. (2025). Pericoronary adipose tissue attenuation predicts compositional plaque changes: A 12-month longitudinal study in individuals with type 2 diabetes without symptoms or known coronary artery disease. Cardiovasc. Diabetol..

[27] Zheng N., Liu Z., Ding Y., Wang X., Li J., Dou G., Xin R., Guo Z., Chen G., Jing J. (2025). Incremental prognostic value of pericoronary adipose tissue attenuation beyond conventional features in patients with nonobstructive coronary artery disease. Atherosclerosis.

[28] Tan N., Marwick T.H., Dey D., Chan W., Nerlekar N. (2025). Association of pericoronary adipose attenuation with major adverse cardiovascular events and high-risk plaque. JACC Cardiovasc. Imaging.

[29] Salem A.M., Davis J., Gopalan D., Rudd J.H.F., Clarke S.C., Schofield P.M., Bennett M.R., Brown A.J., Obaid D.R. (2023). Characteristics of conventional high-risk coronary plaques and a novel CT-defined thin-cap fibroatheroma in patients undergoing coronary computed tomography angiography with stable chest pain. Clin. Imaging.

[30] Mezzetto L., Mastrorilli D., Zanetti E., Scoccia E., Pecoraro B., Sboarina A., Mantovani A., Veraldi G.F. (2024). Clinical risk factors and features on computed tomography angiography in high-risk carotid artery plaque in patients with type 2 diabetes. Int. Angiol..

[31] Zou Q., Qiu T., Liang C., Wang F., Zheng Y., Li J., Li X., Li Y., Lu Z., Ming B. (2025). Multimodal prediction of major adverse cardiovascular events in hypertensive patients with coronary artery disease: Integrating pericoronary fat radiomics, CT-derived fractional flow reserve, and clinicoradiological features. Radiol. Med..

[32] Wang Z., Li Z., Xu T., Wang M., Xu L., Zeng Y. (2025). Long-term prognostic value of computed tomography-derived fractional flow reserve combined with atherosclerotic burden in patients with non-obstructive coronary artery disease. Eur. Radiol..

